# Clustering of Oral Health‐Related Behaviors and Their Association With Sociodemographic Factors in Adolescents Living in England, Wales, and Northern Ireland

**DOI:** 10.1002/cre2.70209

**Published:** 2025-10-30

**Authors:** Ávila‐Oliver Camila, Renato Venturelli, Dominga Ladevig, Tsakos Georgios, Watt Richard G

**Affiliations:** ^1^ Facultad de Medicina Clínica Alemana Universidad del Desarrollo Santiago Chile; ^2^ Department of Epidemiology and Public Health University College London London UK

**Keywords:** adolescents, health behaviors, health inequalities, oral health, United Kingdom

## Abstract

**Objectives:**

This study investigated the clustering patterns of oral health‐related behaviors and their relationship with sociodemographic factors in a national sample of 12‐ and 15‐year‐olds from England, Wales, and Northern Ireland.

**Material and Methods:**

Data from the Child Dental Health Survey (CDHS) 2013 were analyzed. Five individual behaviors were considered: smoking, alcohol use, tooth brushing frequency, sugar intake, and dental attendance. Explanatory variables included sex, age, and eligibility for free school meal (FSM) (as a marker of socioeconomic deprivation). Clustering patterns were assessed using counted clusters, pairwise correlation, and observed/expected ratio analysis. Logistic regression models were performed to assess the associations between behavioral clusters and sociodemographic factors.

**Results:**

The study included 4932 young people, with 51.5% aged 15 years, 50.6% male, and 17.4% eligible for FSM. Statistical differences were found in individual behaviors: Males were more likely to report lower tooth brushing frequency (28.1% vs. 13.6% for females), while 15‐year‐olds were more likely to engage in smoking (28.0% vs. 6.0%) and alcohol consumption (73.5% vs. 29.2%) compared to 12‐year‐olds. FSM‐eligible adolescents were more likely to engage in all risk behaviors, except for alcohol consumption. Seven significant behavioral clusters were identified through O/E analysis, each involving combinations of two or three risk behaviors. In the regression analysis, age was strongly associated with these clusters. For example, 15‐year‐olds had significantly higher odds (OR: 7.04; 95% CI: 4.85–10.22) of exhibiting the cluster involving smoking and alcohol use compared to 12‐year‐olds. Sex and FSM eligibility also showed significant, though weaker, associations with five of the identified behavioral patterns.

**Conclusions:**

Detrimental oral health behavioral clusters were more commonly observed among males, 15‐year‐olds, and adolescents from less advantaged backgrounds.

## 1 Introduction

Oral diseases represent a significant global public health challenge, characterized by their high prevalence, impact on individual quality of life, and chronic nature (Peres et al. 2019). These conditions are closely linked to behavioral choices such as diet, oral hygiene, alcohol consumption, tobacco use, and dental care attendance (Sheiham and Watt 2000). Notably, these health‐related behaviors often cluster together, indicating a pattern rather than isolated habits (Meader et al. 2016; Singh et al. 2013). This clustering is concerning as it increases the risk of multiple chronic conditions, due to the cumulative and synergistic effects of these harmful behaviors (Singh et al. 2013; Austregésilo et al. 2019; Spring et al. 2012). The co‐occurrence of multiple health‐compromising behaviors has been significantly associated with an increased risk of developing diseases, including cancer and cardiovascular conditions (Mesas et al. 2024).

During adolescence, oral health is especially influenced by behavioral patterns. Protective behaviors include regular tooth brushing with fluoride toothpaste, routine dental check‐ups, and maintaining a diet low in added sugars. In contrast, harmful behaviors such as smoking, excessive sugar intake, infrequent dental visits, and poor oral hygiene are associated with an increased risk of dental caries and periodontal disease. These behaviors are shaped by a combination of social, environmental, and individual factors, many of which are established during adolescence (Austregésilo et al. 2019; Alzahrani et al. 2014; Bozzini et al. 2020). Current evidence recommends brushing teeth at least twice daily to maintain optimal oral health (Cury and Tenuta 2014; Marinho et al. 2003). Additionally, a strong correlation has been established between high self‐reported oral health (SROH) scores and positive oral hygiene practices highlighted by literature (Kotha et al. 2017).

Clustered behavioral patterns often emerge in adolescence (Buck and Frosini 2012; Hale and Viner 2016) and frequently involve multiple adverse health‐related behaviors, which tend to be more prevalent among young adolescents rather than older ones (Meader et al. 2016). In addition, behavioral patterns established during adolescence often persist into adulthood (Austregésilo et al. 2019).

During this period of life, oral health is often overlooked, with harmful habits such as poor nutrition, poor oral hygiene, and inadequate access to healthcare becoming increasingly pronounced (Nagaland et al. 2016). Therefore, understanding how young people adopt these health‐compromising behaviors is critical, as these patterns have profound lifelong implications (Brito et al. 2015).

Adolescence, a pivotal developmental stage, is shaped by socioeconomic factors, environmental contexts, and social influences (Viner et al. 2012). These “social determinants of health” play a significant role in adolescents' engagement in health‐compromising behaviors. Moreover, sociodemographic factors are critical in influencing the development of these health‐related behaviors (Currie and Bray 2019).

The purpose of this study was to analyze how different oral health‐related behaviors cluster, explore their associations, and determine how these behavioral patterns are related to demographic and socioeconomic characteristics among young people from England, Wales, and Northern Ireland.

Unlike previous studies that typically examined individual behaviors in isolation or relied on regionally limited datasets, our study applies clustering techniques to a nationally representative adolescent sample. By integrating sociodemographic factors and identifying co‐occurring behavioral patterns, this study offers a more comprehensive and policy‐relevant understanding of adolescent oral health‐related behaviors. This approach fills a critical gap in the literature and can support the development of multi‐behavioral, equity‐focused public health interventions.

## 2 Methods

### 2.1 Study Design and Sample

This study employed a cross‐sectional design based on secondary data from the 2013 Child Dental Health Survey (CDHS), a nationally representative survey of schoolchildren conducted in England, Wales, and Northern Ireland (Anderson et al. 2015).

The survey included 13,628 participants in total. A multistage cluster sampling method was used, with an emphasis on oversampling young people from socioeconomically disadvantaged backgrounds. Additionally, proportionally more students from Northern Ireland and Wales were included to ensure adequate representation relative to the population sizes of these countries. Data collection was carried out through three methods: dental examinations, parent‐completed questionnaires, and self‐administered questionnaires for the 12‐ and 15‐year‐olds, covering various health and lifestyle topics. The self‐administered questionnaire was distributed during school hours under exam‐like conditions. It included both closed and multiple‐choice questions covering oral health practices, general health behaviors, and sociodemographic characteristics. The estimated completion time was approximately 20–30 min.

A detailed description of the sampling and data collection procedures can be found in the CDHS 2013 technical report (Anderson et al. 2015). Ethical approval for the CDHS 2013 was obtained from the Research Ethics Committee at University College London (Project ID 2000/003).

### 2.2 Measurements

For this study, the analysis focused on young people aged 12 and 15, who completed the self‐administered questionnaire.

Relevant measures were selected from the self‐administered questionnaire completed by 12‐ and 15‐year‐olds. Five key oral health‐related behaviors were examined: tooth brushing frequency, smoking status, alcohol consumption, sugar intake, and dental attendance patterns.

Tooth brushing frequency was assessed by a question which asked “how often do you usually brush your teeth?*”* and consisted of six response categories: “more than three times a day,” “three times a day,” “twice a day,” “once a day,” “less than once a day,” and “never.” Hence, this variable was grouped into “twice a day or more” and “once a day or less.” Smoking status was dichotomized into “current/ever smokers” and “never tried smoking cigarettes,” a classification used in previous surveys of both young people and adults (Fuller and Hawkins 2012; Gallagher et al. 2019). To evaluate alcohol consumption, the question “*t*hink about whole drinks (not just sips) when answering this question” was asked, offering five options related to drinking frequency. As with smoking status, this variable dichotomized into “ever/current drinkers” and “never consumed alcohol”. For sugar intake, young people were asked about the frequency of daily consumption of sugary drinks and meals, and the variable was categorized as “four or more times daily” and “less than four times daily,” Finally, dental attendance patterns were dichotomized into “attends dental check‐ups” and “attends only when in trouble or never.”

The primary outcome of the study was the clustering of these health‐related behaviors, which indicate compromised oral health status. The clustering analysis was based on the Observed/Expected (O/E) ratio, providing a quantitative measure of how frequently these behaviors co‐occur more often than by chance. The primary explanatory variables were selected based on their identification as determinants of health‐related behaviors, as outlined by Alzahrani and Tsakos (Alzahrani et al. 2014). This included sex (male or female), age (12‐ and 15‐year‐olds), and socioeconomic position (SEP), assessed through eligibility for FSM. FSM eligibility, a widely recognized indicator of SEP in educational settings (Hobbs and Vignoles 2010), was categorized as “FSM‐eligible” or “FSM‐non‐eligible”. Additionally, SROH was included as a covariate. SROH was dichotomized as “good or very good” and “fair or worse”. Socioeconomic disadvantage was defined by eligibility for FSM, which serves as a widely used proxy for socioeconomic deprivation in UK educational and public health research (Kotha et al. 2017).

### 2.3 Statistical Analysis

Data were analyzed using STATA/IC 12.1 (StataCorp 2011). Appropriate weights were applied to accurately reflect the survey design. A significance level of 0.05 was set for all statistical tests. In describing the sample, all cases were considered, with missing data excluded only in the analytical phase of the study. Associations between missing values, outcomes, and exposures were statistically tested. Chi‐squared test was used to analyze associations between individual behavioral outcomes and explanatory variables. To identify clustering of oral health‐related behaviors, we used three methods: pairwise correlations, counting the number of oral health‐related behaviors, and O/E ratio analysis. Behavioral patterns derived from the O/E ratio analysis were dichotomized as “Yes = 1” and “No = 0”. To examine the association between different patterns of oral health behavior clustering and sociodemographic variables, bivariate and multivariable logistic regression analyses were conducted. Only clusters with an O/E ratio above 1.20 and a prevalence greater than 3% were included in the regression models.

## 3 Results

A total of 4950 young people, aged 12 and 15, were initially selected for the CDHS 2013 survey. Of these, 4932 completed the self‐completion questionnaire. The demographic and socioeconomic characteristics of this sample are presented in Table 1. The sample included a balanced distribution of age and sex. Approximately one in six young people were eligible for free school meals (FSM), reflecting socioeconomic disadvantage. Notably, 70% of participants reported good or very good self‐rated oral health.

**Table 1:** Demographic and socioeconomic characteristics among young people aged 12 and 15 years (*n* = 4932).

Characteristics	*n*	Prevalence %
Age		
12 years	2526	48.5
15 years	2406	51.5
Gender		
Male	2366	50.6
Female	2566	49.4
Free school meal eligibility		
Eligible	1140	17.4
Not eligible	3507	78.5
Missing values	285	4.1
Country		
England	2737	91.1
Wales	1161	5.3
Northern Ireland	1034	3.6
Self‐reported oral health		
Good or very good	3427	70.0
Fair or worse	1476	29.5
Missing values	29	0.5

Table 2 illustrates notable age‐related differences in oral health‐related behaviors. While tooth brushing was generally frequent across both age groups, health‐compromising behaviors—particularly alcohol use and high sugar intake—were more prevalent among older adolescents. A substantial proportion of 15‐year‐olds reported smoking and alcohol consumption, contrasting with much lower figures in the younger group.

**Table 2:** Prevalence of individual oral health‐related behaviors among 12‐ and 15‐years‐olds in England, Wales, and Northern Ireland.

Oral health‐related behaviors	12 years old, *n* (%)	15 years old, *n* (%)
Tooth brushing frequency		
Twice a day or more	1880 (75.3)	1.880 (80.0)
Once a day or less	607 (23.0)	497 (19.3)
Missing data	39 (1.9)	29 (0.7)
Dental Attendance		
For check‐up	2061 (80.0)	1,950 (81.2)
Only when in trouble or never	430 (18.3)	430 (17.5)
Missing data	35 (1.7)	23 (1.6)
Smoking		
Current/Ever smoked	148 (5.8)	691(28.6)
Never smoked	2,316 (91.5)	1,693 (70.0)
Missing data	68 (2.7)	34 (1.4)
Alcohol consumption		
Ever/Current drinkers	666 (26.1)	1,609 (66.6)
Never consumed alcohol	1808 (71.4)	779 (32.2)
Missing data	64 (2.5)	30 (1.2)
Sugar Intake		
High sugar intake	1581 (61.3)	1,506 (59.3)
Low sugar intake	775 (32.3)	818 (37.0)
Missing data	170 (6.4)	82 (3.7)

The analytical sample consisted of 4260 young people who had complete data on all variables used in the regression models. As shown in Table 3, boys were significantly more likely than girls to brush their teeth once a day or less (28.1% vs. 13%, *p* < 0.001). Smoking and alcohol use were strongly associated with age: 15‐year‐olds were far more likely to report both behaviors (28% and 73.5%, respectively) compared to 12‐year‐olds (6% and 29.2%). FSM‐eligible participants consistently exhibited higher prevalence of risk behaviors, including low tooth brushing frequency, high sugar intake, and symptomatic dental attendance.

**Table 3:** Compromising oral health‐related behaviors and their association with sociodemographic factors (*n* = 4260).

	Oral health‐related behaviors				
	Brushing teeth once a day or less, *n* (%)	Dental visits only in trouble or never, *n* (%)	High sugar intake, *n* (%)	Smoking, *n* (%)	Alcohol consumption, *n* (%)
Gender					
Male	618 (28.1)	357 (18.3)	1386 (65.7)	339 (16.3)	1032 (54.5)
Female	339 (13.6)	382 (15.9)	1414 (60.6)	392 (18.8)	988 (50.3)
*p* value	**< 0.001**	0.429	0.129	0.251	0.245
Age					
12‐year‐olds	509 (22.2)	353 (17.3)	1,412 (64.8)	130 (6.0)	583 (29.2)
15‐year‐olds	448 (19.4)	386 (16.9)	1388 (61.6)	601 (28.0)	1437 (73.5)
*p*‐value	0.134	0.831	0.219	**< 0.001**	**< 0.001**
FSM eligibility					
Eligible	264 (27.9)	284 (29.7)	740 (72.4)	223 (22.3)	439 (47.4)
Non‐eligible	693 (19.2)	455 (14.3)	2,060 (61.1)	508 (16.4)	1,581 (53.5)
*p* value	**< 0.001**	**< 0.001**	**< 0.001**	**0.021**	0.117

*Note:* Bold values are statistically significant.

Table 4 presents associations between behavioral clusters and sociodemographic variables. Cluster SA (smoking and alcohol use) was particularly associated with age: 15‐year‐olds were over seven times more likely than 12‐year‐olds to exhibit this pattern (OR: 7.04; 95% CI: 4.85–10.22). FSM‐eligible adolescents also had higher odds of engaging in this cluster compared to their non‐eligible peers (OR: 0.63; 95% CI: 0.44–0.92).

**Table 4:** Association between clustering of health‐compromising behaviors and sociodemographic factors (*n* = 4260).

		Model 1		Model 2		Model 3	
Pattern of clustering	Explanatory variables	OR	95% CI	OR	95% CI	OR	95% CI
Cluster SA	Female (vs. male)	1.24	0.91–1.69	1.25	0.90–1.71	1.26	0.91–1.74
	15‐year‐olds (vs. 12‐year‐olds)	6.76	4.59–9.95*	6.99	4.84–10.12*	7.04	4.85–10.22*
	Non‐FSMs eligible (vs. FSMs eligible)	0.74	0.53–1.05	0.63	0.44–0.90**	0.63	0.44–0.92**
Cluster TD	Female (vs. male)	0.57	0.36‐0.90**	0.37	0.19–0.71**	0.44	0.22–0.89**
	15‐year‐olds (vs. 12‐year‐olds)	3.53	1.88–6.64*	1.01	0.61–1.68	1.15	0.65–2.04
	Non‐FSMs eligible (vs. FSMs eligible)	0.60	0.32–1.14	0.37	0.19–0.69**	0.42	0.22–0.81**
Cluster SAT	Female (vs. male)	0.59	0.36–0.96**	0.57	0.34–0.97**	0.68	0.37–1.24
	15‐year‐olds (vs. 12‐year‐olds)	4.76	2.47–9.19*	4.92	2.57–9.42*	5.53	2.95–10.38*
	Non‐FSMs eligible (vs. FSMs eligible)	0.72	0.37–1.39	0.63	0.34–1.16	0.74	0.39–142
Cluster SAI	Female (vs. male)	1.25	0.84–1.89	1.25	0.80–1.95	1.25	0.81–1.95
	15‐year‐olds (vs. 12‐year‐olds)	4.65	2.91–7.43*	4.91	3.16–7.62*	4.93	3.16–7.67*
	Non‐FSMs eligible (vs. FSMs eligible)	0.57	0.39–0.82**	0.49	0.33–0.71*	0.49	0.33–0.72*
Cluster STI	Female (vs. male)	0.59	0.33–1.05	0.57	0.31–1.06	0.66	0.34–1.29
	15‐year‐olds (vs. 12‐year‐olds)	3.16	1.48–6.74**	3.29	1.55–6.94**	3.59	1.73–1.27**
	Non‐FSMs eligible (vs. FSMs eligible)	0.62	0.32–1.23	0.56	0.29–1.05	0.63	0.33–1.27
Cluster TDI	Female (vs. male)	0.29	0.14–0.58**	0.28	0.15–0.54*	0.34	0.16–0.69**
	15‐year‐olds (vs. 12‐year‐olds)	0.77	0.39–1.55	0.82	0.42–1.61	0.92	0.43–1.98
	Non‐FSMs eligible (vs. FSMs eligible)	0.43	0.21–0.88**	0.42	0.19–0.89**	0.48	0.22–1.05
Cluster SATI	Female (vs. male)	0.57	0.31–1.07	0.56	0.21–1.08	0.65	0.32–1.36
	15‐year‐olds (vs. 12‐year‐olds)	3.88	1.89–7.95*	4.01	1.98–8.15*	4.44	2.26–8.69*
	Non‐FSMs eligible (vs. FSMs eligible)	0.68	0.32–1.44	0.60	0.29–1.21	0.69	0.33–1.42

*Note:* S = smoking; A = alcohol use; T = tooth‐brushing once a day or less; D = dental attendance only in trouble or never; I = high sugar intake.

Model 1 = unadjusted model; Model 2 = mutually adjusted model; Model 3 = model 2 and self‐rated oral health.

* *p* < 0.001

** *p* < 0.05.

In Cluster TD (low brushing frequency and symptomatic dental attendance), girls were significantly less likely to exhibit this pattern than boys (OR: 0.44; 95% CI: 0.22–0.89). A similar trend was observed among non‐FSM‐eligible adolescents (OR: 0.42; 95% CI: 0.22–0.81).

The cluster of smoking, alcohol use, and high sugar intake (Cluster SAI) was significantly associated with age and FSM eligibility. Fifteen‐year‐olds had 4.9 times (95% CI: 3.16–7.67) higher odds of reporting these compromising behaviors compared to 12‐year‐olds. Non‐eligible‐FSM adolescents were 0.49 times (95% CI: 0.33–0.72) less likely to report these behaviors than FSM‐eligible adolescents.

Other behavioral clusters such as SAT and STI also showed strong associations with age, with older adolescents more likely to report these patterns. Cluster TDI (low brushing, symptomatic dental visits, and high sugar intake) was significantly less common among girls, who had 66% lower odds of exhibiting this cluster (OR: 0.34; 95% CI: 0.16–0.69)

Additional analyses revealed behavioral differences based on dental attendance patterns. Adolescents who reported attending routine check‐ups were less likely to smoke, consume alcohol, or report high sugar intake compared to those who visited the dentist only when experiencing problems. They were also more likely to brush their teeth at least twice a day, which aligns with previous findings linking regular dental care with healthier behaviors (Buck and Frosini 2012)

Finally, cluster SATI (smoking, alcohol use, tooth brushing once a day or less, and high sugar intake) was significantly associated with age, with 15‐year‐olds being 4.44 times (95% CI: 2.26–8.69) more likely to report this behavior pattern compared to 12‐year‐olds, after adjusting for covariates including sex, FSM eligibility, and SROH.

## 4 Discussion

In this representative sample of young people from England, Wales, and Ireland, overall behavioral patterns indicated a trend towards positive oral health‐related behaviors, such as regular tooth brushing. However, a significant association between SEP, measured by eligibility for FSM, and individual oral health‐compromising behaviors was observed. Higher proportions of health‐compromising behaviors, such as low tooth brushing frequency, smoking, high sugar intake, and symptomatic dental attendance, were more common among FSM‐eligible adolescents.

The clustering analysis of these health‐compromising behaviors, using the O/E ratio, revealed notable co‐occurrence of detrimental behaviors, especially in clusters involving multiple risk factors. Although clusters of four or five behaviors showed high O/E ratios, they were relatively uncommon, consistent with previous research that has reported low prevalence of multiple oral health‐compromising behaviors (Alamian and Paradis 2009; Lawlor et al. 2005). Clusters involving two or three behaviors, such as smoking, alcohol use and low tooth brushing frequency, were more prevalent but showed lower association, in line with findings that risk behaviors tend to cluster in small groups during adolescence as these patterns begin to form (Austregésilo et al. 2019).

Previous studies have employed the O/E ratio to illustrate clustering of risk behaviors among adolescents (Teh et al. 2019), but comparisons should be made cautiously due to variations in definitions, cut‐off points, and target populations (Alamian and Paradis 2009; Dumith et al. 2012; Sanchez et al. 2007). Nevertheless, the clustering of multiple health‐compromising behaviors observed in this study substantiates findings from previous research involving both adolescents (Alamian and Paradis 2009; Lawlor et al. 2005; Dumith et al. 2012) and adults (Singh et al. 2013; Costa et al. 2013).

Age was a key factor in the clustering of health‐compromising behaviors, with older adolescents being more likely than younger adolescents to engage in clusters involving smoking, alcohol consumption, and other health‐compromising behaviors. This observation is consistent with prior research, indicating that the prevalence of health‐compromising behaviors increases with age during adolescence (Meader et al. 2016; Bozzini et al. 2020; McCabe et al. 2017).

Gender differences were also evident, with females being less likely to participate in clusters of health‐compromising behaviors compared to males. Specifically, girls were less likely to report clusters involving low tooth brushing frequency and symptomatic dental attendance, aligning with previous studies that document sex differences in oral health‐related behaviors (Hiller et al. 2017; Kritsotakis et al. 2016).

The findings of this study also underscore the importance of SEP in shaping behavioral patterns. Adolescents from lower socioeconomic background (FSM‐eligible) were more likely to engage in clusters of detrimental behaviors, such as smoking and alcohol use, further emphasizing the role of social determinants of health (Viner et al. 2012). The clustering of health‐compromising behaviors follows the problem‐behavior theory, which propose that adolescents engaging in one health‐compromising behavior are more likely to participate in others due to the interconnected nature of these behaviors (Jessor 1992).

It is important to note that the data used in this study were collected in 2013. Since then, there may have been shifts in adolescent health behaviors, particularly regarding tobacco and alcohol use, due to evolving public health policies and societal attitudes. Recent governmental reports indicate a decline in smoking among adolescents; however, patterns of sugar consumption and irregular dental attendance remain concerns. Thus, while the findings remain relevant, they should be interpreted in the context of potential behavioral changes over time (NHS Digital 2022; Public Health England 2019).

In the UK, dental care for individuals under 18 is provided free of charge by the National Health Service (NHS), and children are generally invited for annual or biannual check‐ups. However, actual attendance often relies on parental initiative and awareness. Moreover, the extent and quality of oral health education within schools can vary regionally. While many curricula include topics on diet, oral hygiene, and substance use, implementation is inconsistent, particularly in socioeconomically deprived areas. These structural factors may partially explain the higher prevalence of health‐compromising behaviors observed among FSM‐eligible adolescents (Kotha et al. 2017; Gallagher et al. 2019).

Our findings also echo research in adult populations, which identified associations between educational disparities and oral health‐compromising behaviors (Singh et al. 2013). While this study focused on adolescents, it highlights the persistent influence of socioeconomic factors across different age groups. The clustering of smoking and alcohol consumption with other health‐compromising behaviors observed in this study mirror findings in both adolescents and adults (Dumith et al. 2012; Chiolero et al. 2006; Jensen et al. 2003), reinforcing the idea that joint interventions targeting multiple behaviors are more effective than addressing individual behaviors in isolation (Atkins and Clancy 2004).

Although this study benefits from a large, representative sample and robust methodology, certain limitations should be noted. The absence of data from Scotland limits the generalizability of the findings to the entire UK, and the cross‐sectional nature of the study precludes determining causality in the observed relationships. Despite these limitations, the study highlights the need for comprehensive public health strategies that address the interplay between socioeconomic status, age, and sex in shaping adolescent health‐related behaviors. Future research should explore the underlying mechanisms behind these behavioral patterns to guide more effective public health interventions.

## 5 Conclusion

This study found that 15‐year‐olds were significantly more likely than 12‐year‐olds to engage in clusters of oral health‐compromising behaviors. Sex differences also emerged, with females less likely to exhibit these health‐compromising behavioral clusters. Socioeconomic status, measured by FSM eligibility, was strongly associated with these behaviors, with adolescents from lower socioeconomic background more likely to report detrimental behavioral patterns. These findings highlight the importance of age, gender, and socioeconomic factors in shaping adolescent health behaviors and underscore the need for targeted public health interventions to address these inequalities.

## Author Contributions

Conceptualization: Ávila‐Oliver Camila, Watt Richard G, and Tsakos Georgios. Formal analysis: Ávila‐Oliver Camila. Writing – original draft preparation: Renato Venturelli. Writing – review and editing: Ávila‐Oliver Camila, Renato Venturelli, Dominga Ladevig, Watt Richard G, and Tsakos Georgios. All authors have read and agreed to the published version of the manuscript.

## Ethics Statement

This study involved analysis of publicly available or previously collected deidentified data and therefore did not require formal ethics approval.

## Conflicts of Interest

The authors declare no conflicts of interest.

## Data Availability

The data that support the findings of this study are available on request from the corresponding author. The data are not publicly available due to privacy or ethical restrictions.
